# The potential role of stellate ganglion block in impacting the central and peripheral systems: a narrative review

**DOI:** 10.3389/fcvm.2025.1706435

**Published:** 2025-11-20

**Authors:** Zhongjie Zhang, Mingzi An

**Affiliations:** Department of Anaesthesiology and Pain Medicine, Affiliated hospital of Jiaxing University, Jiaxing, Zhejiang, China

**Keywords:** stellate ganglion block, sympathetic nervous system, cardiovascular diseases, cerebrovascular diseases, perioperative stress response

## Abstract

To explore the potential benefits of Stellate ganglion block (SGB) in regulating the central and peripheral systems, as well as its potential as a treatment option for these diseases. We conducted a comprehensive search in PubMed, Web of Science, and Google Scholar libraries using the following keywords: stellate ganglion block, sympathetic nervous system, cardiovascular diseases, cerebrovascular diseases, and perioperative stress response. We selected and critically reviewed research articles published in English related to SGB modulation for the treatment of central and peripheral disease. The collected literature was classified according to content and reviewed in combination with experimental results and clinical cases. SGB can help regulate the cardiovascular and cerebrovascular systems by blocking sympathetic signals, reducing overactivation of the sympathetic nervous system linked to cardiovascular diseases. This local nerve block technique could be a treatment option for these conditions.

## Introduction

Excessive activation of the sympathetic nervous system is considered to be relevant to various central and peripheral system diseases, with the main focus on cardiovascular and cerebrovascular diseases. Resistant hypertension, myocardial ischemia and secretion of brain hormones are due to high sympathetic activity and prolonged activation of the sympathetic nervous system (1–3). The cardiovascular and cerebrovascular systems are regulated by the sympathetic nervous system, parasympathetic nervous system, and sensory nerves, with the sympathetic nervous system playing a pivotal role in disease development (4). Therefore, understanding the modulatory mechanisms of the sympathetic nervous system is crucial to prevent and treat cardiovascular and cerebrovascular diseases. In recent years, researchers have discovered multiple strategies to improve sympathetic nervous activity, such as medication therapy, exercise, diet, and psychological interventions (5, 6). However, the use of medication therapy can lead to side effects, and exercise and dietary interventions demand ongoing dedication to prove their effectiveness. There is currently no optimal intervention to regulate sympathetic nervous activity and reduce the risk of developing cardiovascular and cerebrovascular diseases. Therefore, finding more effective ways to modulate sympathetic nervous system activity remains an important research direction.

Regional nerve block has emerged as a significant area of research due to its effectiveness in modulating regional nerve activity through the inhibition of nerve signals. This technique is extensively utilized in managing various medical conditions and in postoperative pain relief. The Stellate Ganglion Block (SGB) is a traditional approach to nerve blockade, successfully addressing a range of issues, including chronic pain, anxiety disorders, post-traumatic stress disorder (PTSD), and Raynaud's disease (7–9). Ongoing investigations are increasingly highlighting the SGB's potential influence on both central and peripheral nervous systems. Prior research indicates that SGB may impact several cardiovascular parameters, such as heart rate, blood pressure, and vascular function, by altering sympathetic nerve activity (10–12). Specifically, SGB can elicit a cascade of cardiovascular responses, including a reduction in heart rate, peripheral vasodilation, and decreased blood pressure. These physiological changes are deemed significant for enhancing blood flow to the heart and brain, minimizing oxygen demand in myocardial and cerebral tissues, thereby alleviating symptoms associated with cardiovascular and cerebrovascular disorders and potentially improving patient outcomes.

The objective of this narrative review is to present a comprehensive and current analysis of the impact of SGB on both central and peripheral systems, with a specific focus on its mechanisms of action in relation to the cardiovascular and cerebrovascular systems. Additionally, this review aims to offer guidance on clinical assessment and management strategies.

### The stellate ganglion and the sympathetic nervous system

The stellate ganglion, situated along the sympathetic chain of the cervical vertebrae, is formed by the fusion of the seventh cervical ganglion and the first thoracic ganglion, which gives it a characteristic star-like shape (13, 14). It is specifically located beneath the transverse process of the C6 vertebra, adjacent to the carotid sheath. This ganglion primarily contains postganglionic sympathetic neurons that innervate the heart. As components of the peripheral sympathetic nervous system, these neurons facilitate local neural coordination independent of higher brain centers. They influence cardiac function through the release of neurotransmitters such as norepinephrine (5, 15). Consequently, the stellate ganglion is intricately linked to the sympathetic nervous system.

Several researchers have put forth a hypothesis suggesting that the proliferation of cardiac sympathetic nerves and excessive innervation of myocardial nerves may contribute to the onset of ventricular tachycardia, ventricular fibrillation, and sudden cardiac death. Experimental findings indicated that the administration of neurotrophic factors into the left stellate ganglion of canines resulted in increased sympathetic nerve sprouting within the myocardium, with a reported 44% incidence of sudden cardiac death among the treated dogs (3). This hypothesis offers a novel perspective on the management of arrhythmias. In a study examining stellate ganglion activity in a canine model, it was observed that stimulation of the left stellate ganglion could induce ventricular tachycardia and ventricular fibrillation, ultimately leading to myocardial infarction (16). Furthermore, subcutaneous nerve stimulation in dogs with myocardial infarction resulted in remodeling of the stellate ganglion and a decrease in sympathetic nervous activity (17). Additionally, targeted transient potential modulation in the stellate ganglion represents a significant strategy for arrhythmia treatment. The injection of resin toxin into induced transient receptor potential sympathetic neurons for targeted ablation can mitigate ischemia-induced autonomic imbalance and cardiac electrophysiological instability, thereby preventing ventricular arrhythmias associated with acute myocardial infarction (18).

An essential contributor to the progression of heart failure is the heightened activation of the sympathetic nervous system, with the stellate ganglion serving as a component of this system and a potential target for pharmacological intervention. Researchers demonstrated that the injection of nerve growth factor into the stellate ganglion enhanced cardiac norepinephrine reuptake in a rat model of heart failure, thereby alleviating excessive local sympathetic nerve activation in the hypertrophied heart (19). In a chronic heart failure pig model, it was noted that the stellate ganglion exhibited more frequent, transient, and intense co-fluctuations in information processing and cardiac regulation. The neural specificity associated with the cardiac cycle showed considerable variation, and the relationship between neural network activity and cardiac regulation was influenced by the disease state and the degree of co-fluctuations (20). Furthermore, the administration of leptin into the left stellate ganglion was found to stimulate the sympathetic nervous system, resulting in an increased incidence of ischemia-related ventricular arrhythmias (21).

### Application of SGB under ultrasound guidance in clinical practice

SGB is a procedure that entails the injection of local anesthetics into the stellate ganglion, located between the C6 and C7 vertebrae, to inhibit the transmission of signals from the ganglion and modulate the sympathetic nerve fibers it supplies (22). Lidocaine and bupivacaine are among the anesthetics frequently utilized in this technique.

**Figure 1:**
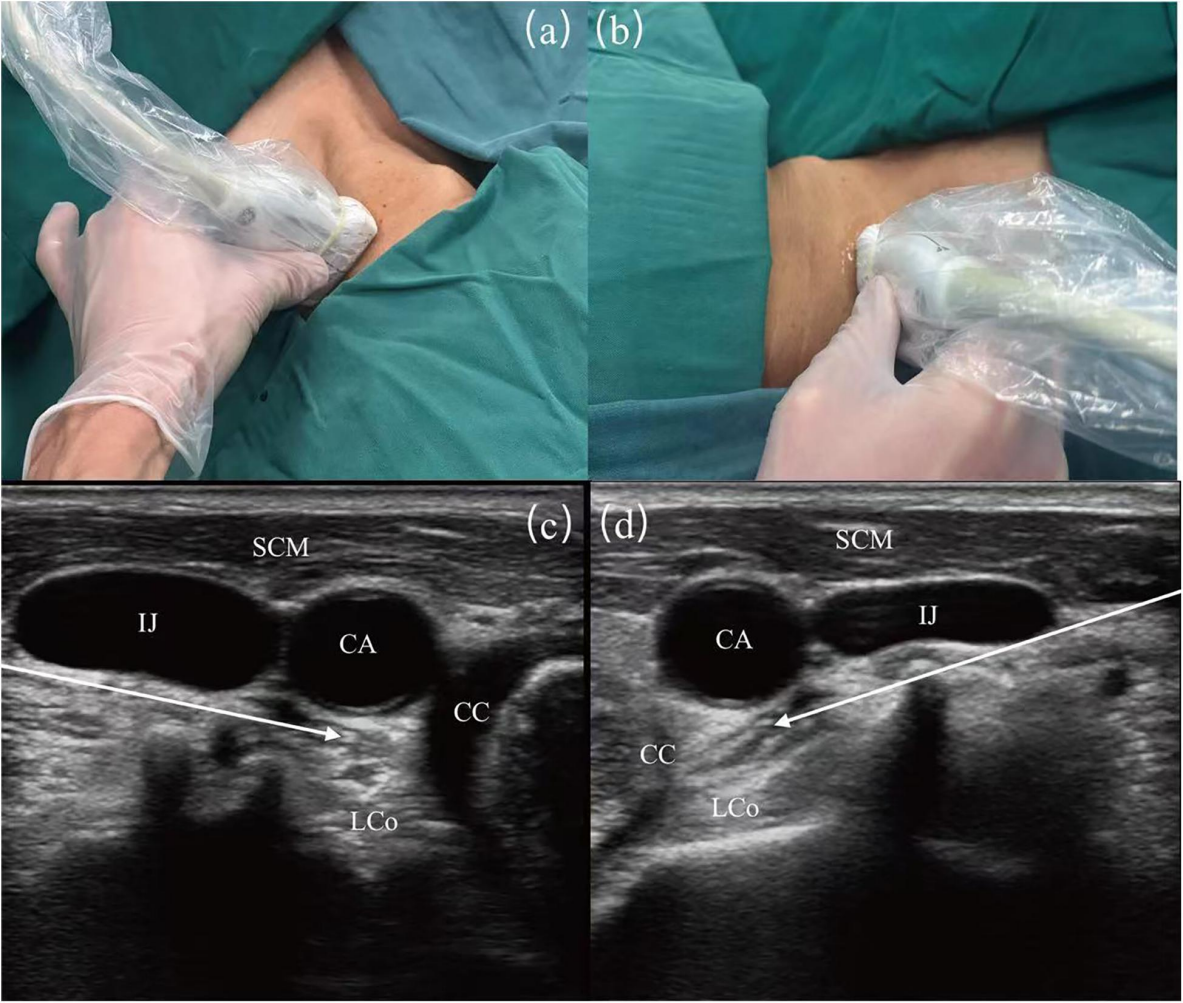
Image guidance for stellate ganglion block (SGB) under ultrasound. **(a)** Surface ultrasound position diagram of left SGB. **(b)** Surface ultrasound position diagram of right SGB. **(c)** Guided diagram for left SGB under ultrasound guidance. **(d)** Guided diagram for right SGB under ultrasound guidance. SCM, sternocleidomastoid muscle; CA, carotid artery; IJ, internal jugular vein; LCo, longus colli muscle; CC, circular cartilage. The white arrows show the path of the puncture needle, with the arrowheads indicating the location of the stellate ganglion.

Techniques described for SGB involve anterior paratracheal, lateral, anterolateral, superior, and posterior approaches. Although SGB have been used in clinical practice for more than 70 years, the literature indicates a variable success rate (16%–100%) (23, 24). With ultrasound guidance, SGB procedures have become more accurate and standardized to ensure the accuracy of injection sites (25). Utilizing ultrasound guidance allows for the accurate identification of the appropriate fascial plane, and positioning the needle at the C6 level facilitates the caudal dispersion of the injectate to the stellate ganglion located at the C7-T1 level. This technique enhances the efficacy and precision of sympathetic nerve blocks while utilizing a reduced volume of injectate. Furthermore, ultrasound-guided stellate ganglion blocks improve procedural safety by allowing direct visualization of vascular structures (e.g., inferior thyroid artery, carotid arteries, vertebral artery, carotid sheath) and soft tissue structures (e.g., thyroid gland, esophagus, nerve roots) (Figure 1) (26).

Despite the fact that ultrasound-guided techniques can greatly enhance the precision and safety of SGB procedures, complications remain an inevitable aspect of clinical practice. Horner's syndrome, the most common complication post-SGB, is now considered a sign of effective blockade. This syndrome presents with miosis (pupil constriction), ptosis (drooping eyelids), enophthalmos (sunken eyes), reduced or absent sweating on the affected side of the face, conjunctival congestion, facial flushing, erythema of the earlobes, nasal congestion, and elevated skin temperature (22). The higher incidence of recurrent laryngeal nerve injury may be associated with the depth of needle insertion. Other associated complications occur less frequently but still necessitate meticulous management. These include transient loss of consciousness, brachial plexus block, high epidural block, and accidental vascular puncture (27). Obstruction of the vertebral artery by the transverse process at the ganglion of the seventh cervical vertebra often hinders clear visualization using ultrasound. Despite the assistance of ultrasound guidance, inadvertent entry of local anesthetics into the vertebral artery poses a serious risk of complications, including sudden cardiac arrest (28).

### Heart

Blocking the stellate ganglion can decrease sinoatrial node discharge frequency, weaken atrioventricular conduction, and raise the ventricular fibrillation threshold due to its close synaptic connections with cardiac nerves. This intervention also tilts the cardiac autonomic nerves balance towards parasympathetic activity, relatively inhibiting sympathetic activity. Consequently, heart rate decreases, myocardial contractility weakens, and myocardial oxygen consumption reduces (29, 30).

SGB also influences ventricular contraction and relaxation. Echocardiogram analysis of 8 healthy individuals revealed a slight impairment in echocardiographic parameters during left SGB-induced ventricular diastole (31). However, this effect is minor and does not hinder ventricular function. Consistent findings from other studies indicate an increase in left ventricular power per beat, cardiac output, and arterial diastolic pressure following SGB administration. Notably, there is no alteration in the systolic or diastolic function of the right ventricle post-SGB (32, 33).

#### Cardiovascular diseases

##### Arrhythmias

The treatment of arrhythmias through cardiac denervation is effective, yet the procedure is intricate and fraught with numerous complications, demanding a high level of expertise from cardiac specialists (34, 35). Transdermal sustained SGB can transiently impede the transmission of sympathetic signals to the heart, presenting itself as a potential therapy for cardiac sympathetic denervation and a viable transition (36, 37). Continuous SGB on the left side is particularly effective for ventricular arrhythmias. A case series study involving two centers included 26 patients with refractory ventricular arrhythmias who underwent percutaneous continuous SGB, with 59% of patients completely suppressing ventricular arrhythmias (38). Similarly, another multicenter retrospective study analyzed 117 patients with refractory ventricular arrhythmias who received SGB treatment, and the results showed that SGB was associated with a reduction in the occurrence of ventricular arrhythmias and the need for defibrillation therapy (39). The left SGB significantly increases the threshold for ventricular fibrillation and prolongs the ventricular effective refractory period (40). This provides a simple, rapid, and effective intervention for patients experiencing electrical storms due to rapid recurrence of ventricular tachycardia or ventricular fibrillation, significantly improving patient outcomes (Table 1).

**Table 1:** Representative studies on SGB apply for arrhythmias.

Trial	Type	Sample number	Intervention	Primary outcome
Savastano et al. (37)	Case	1	Percutaneous SGB	Sustained polymorphic VA
Dusi et al. (38)	Case and Review	26	Continuous percutaneous SGB	VA
Chouairi et al. (39)	Observational	117	SGB	VT and VF
Kim et al. (12)	Observational	89	SGB	HRV
Ouyang et al. (76)	RCT	200	SGB	Perioperative atrial fibrillation
Wu et al. (77)	RCT	90	SGB	Postoperative dysrhythmias
Liu et al. (25)	RCT	100	SGB	Postoperative atrial fibrillation

VA, ventricular arrhythmias; VT, ventricular tachycardia; HRV, heart rate variability.

The impact of SGB on heart rate variability varies depending on the side of the procedure. A study involving 89 head and neck pain patients found no significant changes in heart rate variability index following right-sided SGB. In contrast, left-sided SGB led to an increase in high-frequency range power and a decrease in the low-frequency to high-frequency range power ratio, indicating heightened parasympathetic nervous system activity (12).

##### Myocardial ischemia

Excessive activation of the sympathetic nervous system may play a crucial role in the pathogenesis of vascular calcification. Some researchers have found that SGB can improve aortic calcification in rats by inhibiting endoplasmic reticulum, which is mediated through the suppression of sympathetic activity and norepinephrine release (41). In a study on myocardial infarction models in rats, it was found that SGB significantly reduced the levels of serum cardiac troponin I and troponin T, alleviated ST segment depression and oxidative stress levels. The right-sided SGB was more effective than the left-sided SGB, indicating thaBt SGB can prevent myocardial damage caused by oxidative stress (42).

In the early 20th century, researchers documented a case involving a patient suffering from severe chronic refractory angina who received SGB treatment, followed by a 34-month follow-up evaluation. The findings suggested that repeated SGB may offer significant benefits in alleviating angina, indicating that further investigation into its clinical application is warranted (43). Subsequent research has indicated that SGB can also be effective in treating ventricular fibrillation resulting from myocardial infarction. In one particular case, a patient with an anterior ST-segment elevation myocardial infarction experienced a worsening condition, resulting in loss of consciousness and absence of pulse. After several unsuccessful attempts at defibrillation and pharmacological treatment, SGB was administered, which ultimately restored basic neurological function and hemodynamic stability, enabling successful emergency percutaneous coronary intervention. While this favorable outcome is likely due to multiple factors, it suggests that sympathetic blockade may serve as a potential adjunctive therapy in cases of sustained pulseless ventricular storm (44). Nonetheless, there remains a scarcity of high-quality studies validating the precise efficacy of SGB in human applications.

### Blood vessels

**Figure 2:**
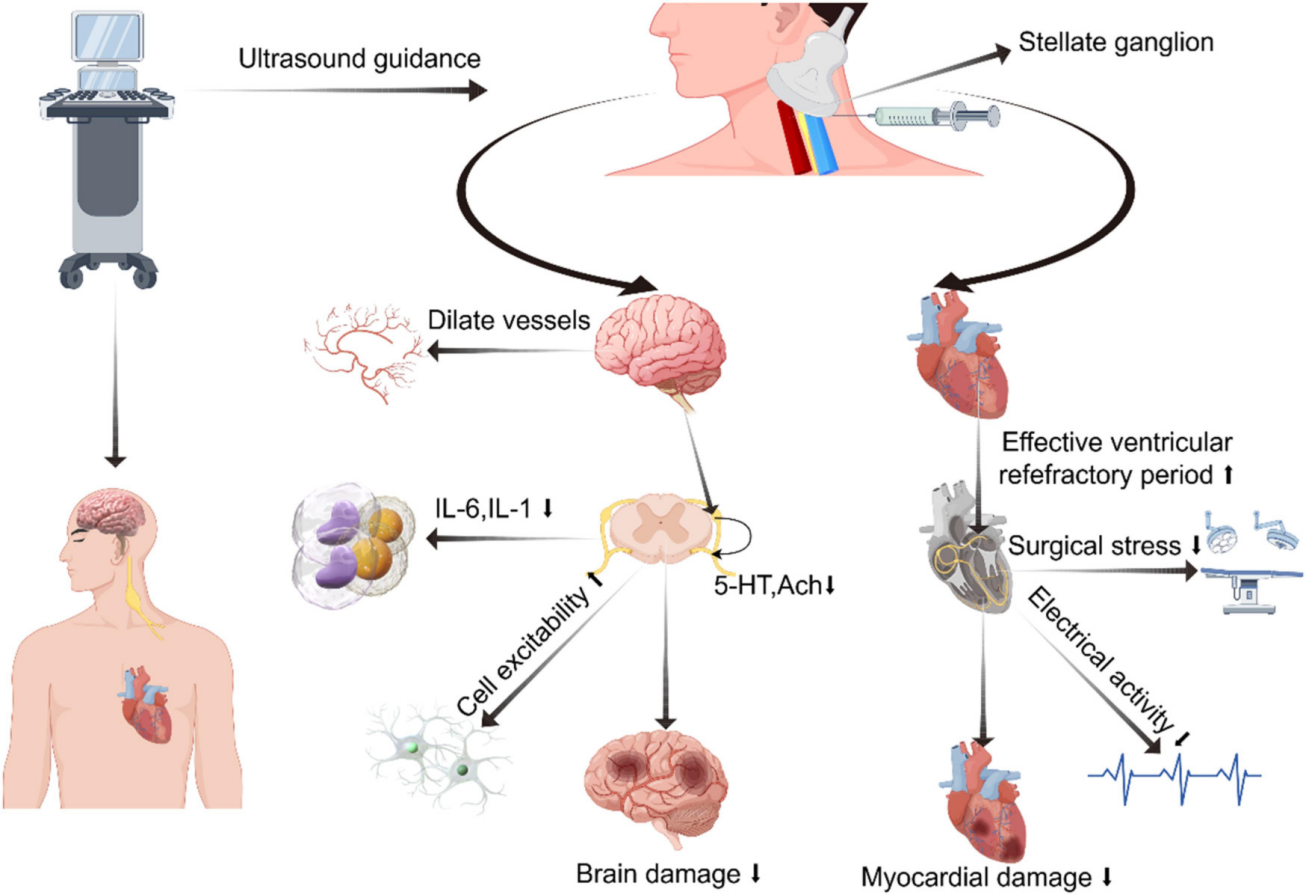
Diagram illustrating potential mechanisms of regulating cardiovascular and cerebrovascular diseases via stellate ganglion block. Created using Figdraw.

SGB also has a certain impact on blood pressure. A study on 16 healthy volunteers showed that after SGB, the reflex sensitivity to stress in patients was reduced within 30 min. This may be due not only to autonomic nervous system imbalance but also to the loss of complexity in heart rate and systolic blood pressure variability (Figure 2) (45). However, a case report has suggested that autonomic nervous system dysregulation post-SGB could induce profound hypertension. This occurrence is thought to stem from the spread of local anesthetics through the carotid sheath, causing vagal nerve inhibition. Consequently, this inhibition reduces baroreceptor reflex sensitivity, prompting a compensatory elevation in sympathetic nervous system function (46).

SGB also causes redistribution of blood flow throughout the body. Research has shown that after performing SGB block in rabbits, blood flow to the lower limbs, internal organs, and the non-blocked side is redistributed to the blocked side (47). A study of 52 orthopedic surgery patients found that Doppler ultrasound assessment showed a decrease in brachial artery resistance index and an increase in blood flow after SGB. However, SGB did not alleviate forearm surgery-related pain (48, 49).

Moreover, recent research indicates that multiple SGB can diminish vasomotor symptoms in female patients (e.g., hot flashes). However, the efficacy of this intervention appears to diminish with prolonged use, underscoring the need for further investigation into its enduring mechanisms of action (7, 50).

### Brain

The protective effect of SGB on the brain remains a topic of debate, yet it is demonstrating a promising trend. In the 1960s, due to the limited technology then, significant changes in cerebral blood flow were not observed in studies of SGB using nitric oxide quantification methods (51, 52). With technological advancements, researchers employ magnetic resonance imaging and direct injection tracking techniques to assess the impact of SGB on cerebral blood flow by measuring blood flow velocity in the neck vessels. Findings indicate a rise in blood flow in the ipsilateral carotid and vertebral arteries primarily attributed to extracranial blood vessel vasodilation (53). After administering SGB to 19 healthy female volunteers, MRI scans revealed a significant increase in the diameter of extracranial arteries, while intracranial arteries showed no change (Figure 2) (54).

#### Cerebrovascular related disorders

Among 23 patients with traumatic brain stroke in a retrospective study, multiple bilateral SGB interventions resulted in a notable increase in overall Neurobehavioral Symptom Inventory scores at one month post-treatment compared to baseline measurements. Notably, male patients demonstrated a more pronounced initial response than their female counterparts (55).

In patients suffering from subarachnoid hemorrhage, SGB has exhibited beneficial effects in protecting the brain. A preliminary analysis of patients with subarachnoid hemorrhage indicated that SGB can alleviate blood flow obstruction in the middle cerebral artery, with effects lasting up to 24 h (56). This suggests that SGB possesses a notable vasodilatory effect on cerebral vasospasm. Similar studies have confirmed these findings (57).

Another randomized controlled study involving 102 patients with subarachnoid hemorrhage showed that SGB reduced levels of early brain injury markers within 7 days postoperatively, including IL-1β, IL-6, TNF-α, ET-1, NPY, NSE, and S100β. Additionally, the increase in blood flow in the middle cerebral artery and basilar artery was reduced (58).

#### Original neurological diseases

Several researchers have conducted preliminary investigations on the regulatory role of SGB in preserving brain function within the neuroendocrine-immune network, with a focus on 50 patients who experienced traumatic brain injury. Post-SGB treatment, a notable decline was observed in IL-6, IL-1β, TNF-α, and NF-κB p65 proteins levels in comparison to the control group. Notably, NF-κB p50 protein levels showed no change, indicating a potential modulation by SGB in neuroendocrine-immune system dysfunction post- traumatic brain injury (TBI) (59). Studies conducted earlier have suggested that SGB can change the distribution of lymphocyte subpopulations and NK cell activity, even if only temporarily (60). After a TBI, dysfunction in the autonomic nervous system of the heart may occur, characterized by increased cell excitability in stellate ganglion neurons and decreased excitability in intracardiac ganglion neurons. This alteration in peripheral cardiac efferent neuron function is thought to be influenced by the modulation of transient A-type K + currents and/or M-type K + currents (61).

SGB has been employed in central post-stroke pain patients, yielding positive results. A case study confirmed the analgesic efficacy of this intervention in a 67-year-old individual experiencing severe paroxysmal spasm-like pain in the right side of the head, upper, and lower limbs due to intractable central post-stroke pain. patient's pain levels decreased after receiving SGB for 7 days, and during a follow-up 9 months later, the patient was found to be pain-free (62).Other researchers have reached comparable conclusions as well (63). Furthermore, SGB has been found to effectively lessen thalamic pain syndrome. Following SGB treatment, a significant reduction in headache, facial, and upper limb pain on the affected side was noted in 2 patients with thalamic pain syndrome (64). Currently, only case reports on central pain are available, underscoring the necessity for additional randomized controlled trials to investigate the mechanism of action of SGB.

### Others

SGB is widely utilized in the treatment of various diseases due to its simplicity and minimal risk. Initially intended for alleviating chronic pain syndromes related to the sympathetic nervous system (65, 66), SGB has been shown in previous studies to reduce postoperative opioid consumption in patients undergoing upper limb orthopedic surgery under general anesthesia (67) Clinical trials have shown that SGB alleviates neuralgia in patients with herpes zoster affecting the face and head. After six sessions, patients reported a notable decrease in their VAS pain score, although further investigation is required to elucidate the precise mechanism of action (68).

A prospective study on military personnel demonstrated that SGB reduces anxiety symptoms in PTSD patients and enhances their quality of life (9). SGB has also been shown to improve sleep quality in individuals with sleep disorders. A study following breast cancer patients for 24 weeks revealed that the likelihood of experiencing improved sleep quality was 3.4 (95% CI 1.6–7.2) at week 1 and 4.3 (95% CI 1.9–9.8) at week 24 compared to the beginning (7). By modulating hormone levels in the hypothalamic-pituitary-adrenal axis related to sleep activity, SGB serves as an alternative therapy to sedatives, potentially enhancing postoperative sleep quality and overall recovery (69).

SGB provides initial advantages for gastrointestinal function as well. Patients with ulcerative colitis who received SGB showed a notable decrease in inflammatory markers within a month, indicating a possible association with SGB's modulation of neuroimmune pathways (70).

#### Perioperative responses

The role of SGB is important in perioperative cardiovascular management. A study conducted with 20 patients undergoing knee arthroscopy demonstrated that preoperative SGB can mitigate tourniquet-induced hypertension and help stabilize hemodynamic parameters (71). Anesthesiologists have consistently faced a formidable challenge in managing the stress response triggered by endotracheal intubation following anesthesia induction. During a study involving 60 elderly patients slated for elective surgery, it was observed that SGB decreased blood pressure and heart rate reactions post-tracheal intubation, thereby alleviating intraoperative stress responses (72). Similarly, another investigation revealed that preoperative SGB mitigate stress responses during carbon dioxide insufflation in elderly patients, thereby stabilizing hemodynamic parameters (73). Furthermore, a study involving 80 patients undergoing elective surgery for coronary heart disease demonstrated that preoperative SGB could decrease both the frequency and intensity of symptoms associated with coronary heart disease, consequently reducing the overall risk for these patients (74).

Cardiovascular events are more prone to occur in thoracic surgery due to its proximity to the heart, the inflammatory response, incision pain, and mechanical traction. A study on visceral pain in elderly patients undergoing thoracoscopic lung cancer surgery revealed that preoperative SGB reduces intraoperative visceral stress, stabilizes hemodynamics during chest incision, and alleviates visceral pain within 24 h post video-assisted thoracoscopic surgery (75).

A study of 200 postoperative lung resection patients receiving SGB prior to surgery demonstrated a notable 7% reduction in atrial fibrillation incidence, as determined through dynamic electrocardiogram analysis conducted during and 24 h post-surgery (76). A separate study involving 90 patients who underwent lung resection revealed that preoperative SGB could decrease the occurrence of postoperative supraventricular tachycardia by around 20% within 48 h following surgery, while also substantially extending the duration of postoperative sleep for the patients (77). An analysis of 50 patients demonstrated that SGB did not have a significant effect on lung function (78), thus ensuring the safety of thoracic surgery for these patient.

Moreover, there is a lack of research investigating the potential of SGB to mitigate perioperative myocardial ischemia and injury, thereby decreasing the occurrence of perioperative myocardial infarction. Some scholars have published a study protocol regarding the occurrence of myocardial injury following laparoscopic radical resection of rectal cancer. However, this study reported no instances of myocardial injury during surgery following the administration of SGB postoperatively (79). The clinical value of SGB in perioperative cardiovascular management may be attributed to its capacity to modulate sympathetic nervous activity, improve hemodynamics, and reduce oxidative stress. Consequently, SGB presents potential clinical efficacy in perioperative cardiovascular management.

## Conclusion

In this narrative review, we have summarized the modulating effects of SGB on the central and peripheral focus on cardiovascular systems and related diseases. SGB blocked the transmission of sympathetic nerve signals to alleviate various cardiac arrhythmias and cardiovascular diseases. At the same time, it also reduced cardiac and cerebral infarction and primary diseases through anti-inflammatory pathways and inhibiting oxidative stress. Additionally, the narrative detailed the influence of SGB on stress responses and multiple postoperative outcomes among surgical patients.

Nonetheless, SGB also produced vagus nerve stimulation effects, encompassing insomnia, depression, and cognitive impairment, in addition to alleviating pain. Perhaps it would be beneficial to refocus our inquiry on whether SGB can produce effects resembling those of the parasympathetic nervous system, a subject requiring further investigation. Therefore, high-quality clinical studies studies are needed to support the administration of SGB for the central and peripheral systems.
